# Bidding for Pauper Practice

**Published:** 1878-06

**Authors:** John Wright

**Affiliations:** Clinton, Ill.


### BIDDING FOR PAUPER PRACTICE.

*To the Editor of the Journal and Examiner:*

I desire to offer, with your permission, a few comments on some of the points alluded to under the head of "Remarks," in the March number of the *Chicago Medical Journal and Examiner*, in reference to bidding for pauper practice by physicians. Among others, the following question is asked: "Is it proper to bid for pauper practice?" and answered as follows: "That is a matter of taste." I confess that your response to that question rather surprised me. I think the word, bad, should have been appended to the word taste, so that the answer would read: It is in bad taste for physicians to bid for pauper practice. Such a response would be, I think, in harmony with the code of ethics, which is the expressed public sentiment of the medical profession, and also in harmony with the decision of the Judicial Council on this very question.

In Vol. XX of the Transactions, we find the following language: "Whereas, The contract system is contrary to medical ethics, *Resolved*, That all contract physicians, as well as those guilty of *bidding for practice* at less rates than those established by a majority of regular graduates of the same locality, be classed as irregular practitioners." This decision of the Judicial Council is the decision of the American Medical Association, and that decision, and the assertion that it is a mere matter of taste with physicians whether or not they bid for practice, do not harmonize well. In fact I think there is a direct conflict between them.

"Is it a violation of the code?" The Judicial Council, Vol. XX, page 41, also answers that question, and I will say nothing on that point.

"The physicians of every district shall establish a fee bill, and make it a point of honor to adhere to it." The first part of this quotation will be found, by reference to the code of ethics, to belong to and to be taken from some other document than the code of ethics. The code does not say that physicians shall establish a fee bill. There is no such declaration in the code of ethics. The language of the code on the subject, is that of a suggestion merely and nothing else. Such words as fee bill, cannot be found in the code, and as the remarks upon this subject were made from an erroneous stand point and outside of the question in dispute, I will say nothing further in this connection at this time, as the simple allusion to the question and the code is deemed all that is necessary to show the error.

I also differ with you in regard to who it is that make up the list of bidders for the pauper practice. My observation leads me to believe that the list is not made up from the ranks of the recent graduates, or from the ranks of those that are poor; for I notice that in localities where bidding for practice is tolerated by the profession, the old as often as the young physicians and the rich as often as the poor, are found on the list of bidders, which leads me think that those who bid for practice are not driven to it from necessity in order to keep their families or themselves from starvation, but I believe it is due, as a rule, to a disregard of that professional honor that should be found in every man that belongs to the profession. These men forget or disregard the fact that they are professional men as well as business men, and that there is such a thing as a dishonorable business. I can see no difference between bidding for pauper practice and bidding for the practice of individuals or families. That which prompts the man to bid is the same in all cases.

In my opinion, all bidding for practice, by physicians against each other, whether it be for pauper practice or the practice of individuals or families, is alike an open violation of the spirit of the code of ethics, and contrary to the plain decision of the American Medical Association, as expressed by the Judicial Council. I am sorry that there are so few that are willing, or who feel called upon to speak out against competitive bidding for practice; and also sorry to see any defend the practice, or touch the subject so feebly as to lead the advocates of bidding to construe their remark into a defense and justification of those that enter into competitive bidding for practice.

If the code of ethics is wrong or faulty, let that document be changed, but let us not disregard or openly evade it while it is the acknowledged code of rules and principles to guide us, and is believed to be all that is necessary if observed, to promote harmony and good feeling among physicians, and also between the latter and the public. The question is not whether the code is the best that could be devised, but whether bidding for pauper practice is in harmony with the code as it now stands, or is a violation of it.

Respectfully yours, JOHN WRIGHT.

CLINTON, Ill., May 6, 1878.

REMARKS.—We are pleased to publish the above communication, because it affords us an opportunity to define more clearly the views we expressed on a previous occasion. The resolution of the Judicial Council in reference to bidding for practice, is so clear that there can be no doubt of its meaning, and we could have answered the question, "Is it a violation of the code?" simply by referring to this decision of the American Medical Association. But we wished to explain why this decision must be a dead letter. It tries to make the impossible, possible; to assume control over circumstances which are entirely beyond its reach. Suppose a workman with a large family needs a physician for an attack of sickness which requires daily medical attendance for five weeks. He is willing to pay a moderate fee, but unable to meet a bill of seventy dollars at the rate of two dollars a visit. Suppose the first physician the workman wishes to employ, strictly adheres to the customary fee, and declines attendance at a less rate, while another takes the case at one dollar per visit. Is this action of the second physician honorable or dishonorable? The case would assume a different phase if the first physician were employed at the regular rate, and the second tried to obtain the case by bidding below the former. This would be considered a mean and dishonorable act, even without a code of ethics.

We did not wish to defend any violation of the code, but we tried to show to our correspondent that an appeal to this code will not stop the practice of bidding he is so anxious to abolish. And we suggested to him a plan which, in our opinion promises better than the dictation of the Judicial Council. He should get his professional friends to unite their efforts with his in exposing the absurdity of a system which invites bids for medical services and lets the pauper practice to the lowest bidder in the same way as contracts for fuel are let. Corporations should establish a fixed salary for him who engages to attend paupers; then they will not have to select from inferior men, but from the best among all competitors.

In conclusion, we have to admit that our correspondent is right in what he says with reference to the words: "The physicians of every district should establish a fee-bill," etc. This quotation is taken from another document, and does not reproduce strictly the *letter* of the code—but it nevertheless expresses its *spirit*.—Ed.

